# The Application of Probiotic Bacteria from Strawberry (*Fragaria ananassa* × Duch.) in the Fermentation of Strawberry Tree Fruit (*Arbutus unedo* L.) Extract

**DOI:** 10.3390/microorganisms12051000

**Published:** 2024-05-15

**Authors:** Deni Kostelac, Filip Dolenec, Anica Bebek Markovinović, Ksenija Markov, Danijela Bursać Kovačević, Jadranka Frece

**Affiliations:** Faculty of Food Technology and Biotechnology, University of Zagreb, Pierottijeva 6, 10000 Zagreb, Croatia; dkostelac@pbf.hr (D.K.); filipdolenec1@gmail.com (F.D.); anica.bebek.markovinovic@pbf.unizg.hr (A.B.M.); kmarkov2@pbf.hr (K.M.); danijela.bursac.kovacevic@pbf.unizg.hr (D.B.K.)

**Keywords:** probiotics, fermentation, strawberry, strawberry tree fruit, functional food

## Abstract

The search for unexplored plant resources that would provide a good basis for the development of novel probiotic functional foods is rapidly increasing. In this context, the strawberry tree fruit (*Arbutus unedo* L.) is particularly interesting, as it is rich in numerous antioxidant bioactive compounds that have been shown to be beneficial to health, but have not yet found industrial applications. In this work, the probiotic characterization of lactic acid bacteria strain *Lactiplantibacillus plantarum* DB2, isolated from strawberries (*Fragaria ananassa* × Duch.), was performed. The tested strain proved to be safe to use, displaying no antibiotic resistance or hemolytic activity. Due to its proven probiotic potential during simulated gastrointestinal transit, its antimicrobial activity, and its coaggregation with pathogens, it was selected for fermentation of an aqueous *Arbutus unedo* L. extract, which was subsequently microencapsulated and freeze-dried to extend its shelf life and preserve its functional properties. The antioxidant activity of the ferment obtained was maintained (80%), while after microencapsulation and freeze-drying, about 50% and 20% of the antioxidant activity was retained, respectively. In conclusion, this study demonstrates for the first time the application of probiotics isolated from strawberries in the fermentation of strawberry tree extract and monitors the antioxidant activity during post-fermentation formulation, paving the way for a potential industrial application of this underutilized plant.

## 1. Introduction

In recent years, research regarding natural sources of bioactive compounds (BACs) with potential health benefits has attracted considerable attention in the field of food science and nutrition. Among these sources, berries and medicinal plants have emerged as promising reservoirs of BACs [1], with a variety of properties that contribute to human health and well-being [2]. In this context, strawberry (*Fragaria ananassa* × Duch.) and the strawberry tree (*Arbutus unedo* L.) have attracted great interest due to their high content of antioxidants and other health-promoting compounds [3,4].

Underutilized plant resources, such as the strawberry tree fruit (*Arbutus unedo* L.), are increasingly becoming the focus of exploitation, in this case, mainly for its medicinal properties, with particular attention to its antioxidant-rich extract [5]. Extracts from *A. unedo* have long been used in folk medicine and also in modern pharmacotherapy for their ability to combat oxidative stress and inflammation [6]. In times of global crisis, there is an increasing demand for new functional foods that provide consumers with an adequate intake of all nutrients and BACs in a simple and safe way. Recently, the possible addition of probiotics from plant extracts has been investigated to provide functionality and added value to foods, in addition to their usual nutritional value [7]. 

The term “probiotic” refers to mixtures of one or more bacterial species that have a positive effect on the health of the host to which they are administered by improving the characteristics of its intestinal microflora [8,9]. The most common beneficial effects of probiotics are the lowering of cholesterol levels; stimulation of the immune system; increased absorption of minerals; and antimicrobial, anti-carcinogenic, and antihypertensive effects [10]. Probiotic bacteria are conventionally incorporated into fermented dairy products. However, their use in such matrices is often hindered by lactose intolerance or dietary restrictions that necessitate cholesterol reduction. A potential solution to these limitations lies in plant extracts, emerging as a novel carrier category for probiotic bacteria. They offer the advantage of providing a suitable nutrient content for bacterial growth, thereby stabilizing the probiotic product [7]. The use of probiotics improves the nutritional properties of the product, while the addition of probiotics in plant extracts increases the antioxidant activity, specific to certain strains of lactic acid bacteria [11,12]. The selection of the right bacterial strains for the production of fruit preparations is crucial, as achieving their stability, survival, and functionality is more challenging than is the case for fermented dairy products [13]. 

Probiotic bacteria such as *Lactiplantibacillus plantarum* have been extensively studied for their positive effects on gut health and general well-being. These bacteria, found primarily in fermented foods, have been associated with improved digestion, enhanced immune function, and a balanced gut microbiota [14]. Recent research highlights the great potential of *Lactiplantibacillus plantarum* in inhibiting fungal growth, which plays a crucial role in the elimination of mycotoxins, greatly expanding the possibilities for their application in the food and pharmaceutical industries [15].

Combining these probiotic strains with the antioxidant-rich compounds from strawberries and strawberry tree fruit extract could potentially enhance their individual health benefits, creating a unique symbiotic relationship that addresses both gut health and oxidative stress. 

Therefore, the aim of this study was to investigate the probiotic potential of lactic acid bacteria strains isolated from strawberries. Furthermore, the synergistic effect of the combination of the best probiotic candidate strain with an extract from the strawberry tree fruit was investigated. Extract of *A. unedo* was fermented with a probiotic strain, and the resulting ferment was microencapsulated and freeze-dried. By evaluating the antioxidant and antimicrobial potential of this combined approach, we aim to elucidate whether the integration of probiotic bacteria with natural extracts can lead to a more potent and efficient strategy to promote health and prevent diseases related to oxidative stress.

## 2. Materials and Methods

### 2.1. Chemicals and Reagents

All reagents and solvents used for the analyses were of analytical grade. Pepsin was obtained from Sigma Aldrich GmbH (Taufkir-chen, Germany), salts (NaCl, KCl, NaH_2_PO_4_, Na_2_SO_4_, NaHCO_3_, CO(NH_2_)_2_, CaCl_2_), ethanol, and hydrochloride acid were obtained from Kemika (Zagreb, Croatia). DPPH was obtained from Merck KgaA (Darmstadt, Germany).

### 2.2. The Fruit Samples 

The strawberry fruits (*Fragaria × ananassa* Duch., cv. ‘Albion’) were procured via the company Jagodar-HB d.o.o (Donja Lomnica, Zagreb, Croatia). Upon arrival at the laboratory, the stems were removed from the fruits, and the fruits were washed with tap water, wiped with a sterile cloth, packed in plastic bags, and stored at −18 °C. For the isolation of lactic acid bacteria (LAB), the strawberry fruits were thawed at room temperature.

The strawberry tree fruits (*Arbutus unedo* L.) were harvested on the island of Mali Lošinj, Croatia, and immediately transported to the laboratory. The fruits were washed, dried, packed in plastic bags, and stored at −18 °C. For the extraction process, the strawberry tree fruits were thawed at room temperature, crushed, and homogenized with a household blender to obtain a pulp.

### 2.3. Isolation, Identification, and Cultivation of Bacterial Strains

The strawberry fruits were thawed at room temperature, and 5 g of the fruits were aseptically separated and transferred to a 200 mL flask in 95 mL of sterile physiological solution (0.85%) containing glass beads. The prepared suspension was shaken in a KS 4000i shaker (IKA-Werke GmbH & Co. KG, Staufen, Germany) at 150 rpm for 30 min to break up the fruit pieces and release the microorganisms from the pulp.

After mixing, decimal dilutions of the suspension were prepared, inoculated on MRS agar (Biolife, Milan, Italy), and incubated at 37 °C for 24–48 h. Afterwards, individual milky-white colonies of round shape were observed on MRS agar (Biolife) from the undiluted original sample. These colonies were subcultured onto MRS agar using the streaking method and incubated again at 37 °C for 48 h.

Successfully isolated pure cultures were microscopically analyzed, inoculated into MRS broth, incubated at 37 °C for 48 h, and preserved with the addition of 30% glycerol at −20 °C and −80 °C for later use.

After overnight cultivation on MRS agar, the selected isolate was identified using the API 50 CHL test (BioMerieux, France), as per the manufacturer’s instructions. The results were interpreted using the Api-webTM identification software (https://apiweb.biomerieux.com/login, access on 10 October 2023) (bioMérieux, Marcy-I’Étoile, France).

Total genomic DNA was isolated according to the procedure described by Martín-Platero et al. [16], serving as a template for polymerase chain reaction (PCR) using Q5 polymerase (NEB, Ipswich, MA, USA), following the manufacturer’s instructions. Sequences encoding 16S rRNA were amplified and sequenced with the previously described primers 27F and 1492R [17]. RecA loci were amplified and sequenced with the designed primers LbrecA-f (5′-TTGGCTGATGCACGGAAA-3′) and LbrecA-r (5′GCGAGGATTATACCGAAAACATTCAT-3′), which amplify the recA gene in *Lb. plantarum* [18], *Lb. paraplantarum*, and *Lb. pentosus*. The obtained PCR products were purified using the Monarch DNA gel extraction kit (NEB, USA) and then sequenced. The DNA sequence was analyzed by searching the EzBioCloud database and the NCBI BLAST database [19].

The bacterial strains were cultivated in MRS broth under aerobic conditions at 37 °C for 24 h. After incubation, the bacterial cells were separated from the medium by centrifugation at 6440× *g* for 10 min. After centrifugation, the cells were resuspended in sterile deionized water and centrifuged again under the same conditions. After rinsing, the cells were resuspended in a specific volume of sterile deionized water. The prepared suspensions were homogenized and used for the subsequent experiments. 

### 2.4. Probiotic Characterization of Bacterial Isolates

#### 2.4.1. Survival of Probiotic Candidates in Simulated Gastrointestinal Fluids

The degree of survival of the isolated bacterial strains under simulated gastric and intestinal conditions was determined using solutions prepared as described previously, with minor modifications [20]. Simulated gastric juice was prepared by suspending pepsin (3 g/L) in a solution containing the following salts: NaCl (9 g L^−1^), KCl (0.8946 g L^−1^), NaH_2_PO_4_ (0.8878 g L^−1^), Na_2_SO_4_ (1.680 g L^−1^), NaHCO_3_ (1.680 g L^−1^), and CO(NH_2_)_2_ (0.1981 g L^−1^). The pH of the solution was adjusted to 3 using concentrated hydrochloric acid. Simulated small intestinal juice was prepared by suspending pancreatin (1 g L^−1^) and porcine bile salts (Merck KgaA) (3.0 g L^−1^) in a solution containing the following salts: NaCl (9 g L^−1^), KCl (0.8946 g L^−1^), NaH_2_PO_4_ (0.8878 g L^−1^), Na_2_SO_4_ (1.680 g L^−1^), NaHCO_3_ (1.680 g L^−1^), and CO(NH_2_)_2_ (0.1981 g L^−1^). The pH of the solution was adjusted to 7.09 with concentrated hydrochloric acid. The tested strains were exposed to simulated gastric conditions for 2 h at 37 °C, separated by centrifugation (6440× *g*, 10 min), and resuspended in simulated intestinal solution for 4 h at 37 °C. The value of the colony-forming unit (CFU) was determined using the plating method on MRS agar after a 24 h incubation at 37 °C.

#### 2.4.2. Antimicrobial Activity of *Lpb. plantarum* DB2 against Pathogenic Bacteria

The bacterial cells of *Lpb. plantarum* DB2 were cultivated overnight in MRS broth (Biolife) at 37 °C and separated from the medium by centrifugation at 6440× *g* for 10 min. The resulting supernatants were filter sterilized using 0.22 µm syringe filters (ISOLAB Laborgeräte GmbH, Eschau, Germany), their pH was measured with a pH-meter (Mettler Toledo, Columbus, OH, USA), and they were used for the subsequent determination of antimicrobial activity.

The antimicrobial activity of the obtained supernatants was investigated using a modified technique, according to the method of Ratsep [21], using 96-well polystyrene plates against the following test microorganisms: *Escherichia coli* ATCC^®^25922^TM^, *Listeria monocytogenes* ATCC^®^23074^TM^, *Staphylococcus aureus* ATCC^®^25923^TM^, and *Salmonella* Typhimurium ATCC^®^29631^TM^. After overnight cultivation of test microorganisms in nutrient broth (Biolife) at 37 °C for 24 h, the cells were harvested by centrifugation and resuspended in nutrient broth with a McFarland density of 3.0. Volumes of 200 µL of the tested supernatant, 70 µL of the nutrient broth, and 10 µL of the pre-cultured test organism were pipetted into the wells of the 96-well microtiter plates. The plates were incubated for 24 h at 37 °C under aerobic conditions. The ability to inhibit growth was determined using the absorbance measurements at 620 nm, before and after incubation, according to Formula (1):

Inhibition (%) = (1 − A_t_/A_0_) × 100 (1)

where A_t_ is the absorbance at time t, and A_0_ is the absorbance at time 0.

Instead of supernatant, the control samples contained uninoculated MRS broth, but were treated the same as the other samples, and the samples without the addition of pathogenic microorganisms were the blanks.

#### 2.4.3. Antibiotic Resistance Assay

The determination of bacterial sensitivity to selected antibiotics was performed according to the guidelines of the European Committee on Antimicrobial Susceptibility Testing (EUCAST). The method was slightly modified to adapt to the tested strain.

Bacterial culture of *Lpb. plantarum* DB2, grown in MRS broth, was separated from the medium by centrifugation at 6440× *g* for 10 min and resuspended in sterile water, with OD values adjusted to 0.5 ± 0.05. The cell suspension was applied to MRS agar plates by uniformly swabbing a cotton swab, previously immersed in the suspension, in three directions. After inoculation, discs of the tested antibiotics (Liofilchem S.r.l., Roseto degli Abruzzi, Italy) were placed on the inoculated MRS agar. The plates were then incubated at 37 °C, and after 24 h, the appearance of inhibition zones was observed, indicating the sensitivity of the tested strain to the antibiotic. 

In this study, the sensitivity of lactic acid bacteria isolate DB2 to the following antibiotics was investigated: ampicillin (10 µg), erythromycin (15 µg), gentamicin (10 µg), kanamycin (30 µg), clindamycin (10 µg), chloramphenicol (10 µg), streptomycin (10 µg), tetracycline (30 µg), and vancomycin (30 µg) (Liofilchem S.r.l., Roseto degli Abruzzi, Italy).

#### 2.4.4. Hemolytic Activity

The hemolytic activity of the lactic acid bacteria isolate was determined according to the method described by Halder et al. [22]. Bacterial cultures grown overnight in MRS broth were inoculated onto Columbia blood agar and incubated at 37 °C for 24 to 48 h. After incubation, the presence or absence of hemolytic zones on the blood agar was observed.

#### 2.4.5. Biofilm Formation Ability

The ability to form biofilms was determined using a modified method described by Borges et al. [23]. The method is based on the classification of the strength of the biofilms formed in polystyrene microtiter plates after incubation.

Briefly, 3 mL of MRS broth was added to 12-well polystyrene microtiter plates and inoculated with 100 µL of a suspension of previously cultured bacterial cultures. The plates were then incubated at 37 °C for 24 h or 48 h. The contents of the wells were then carefully pipetted out. The bacterial cell sediment was washed with 2 mL of sterile deionized water, and 2 mL of methanol was added to fix the cells adhering to the biofilm. The adherent cells were stained by adding 1% crystal violet for 5 min. Excess color was removed by thorough rinsing the cells with deionized water, and the bound color was released by adding 2 mL of 33% acetic acid. The optical density (OD) was then measured using a spectrophotometer at 595 nm. Non-inoculated samples were used as negative controls.

The obtained optical density values were compared with the optical density of the negative control, and the biofilm-forming ability was categorized as weak, moderate, or strong, according to the specifications of Borges et al. [23].

#### 2.4.6. Autoaggregation and Coaggregation of LAB Strain

The degree of autoaggregation was determined according to the methods of Kos et al. [24], with a modification of the method of Kostelac et al. [25]. The measurement of the absorbance (at 620 nm) of the upper layer of the suspensions, modified to OD values of 0.5 ± 0.05 to standardize the number of bacterial cells, which remained stationary for 24 h, was carried out at intervals of 1, 2, 3, 4, and 24 h. The autoaggregation rate was calculated using the following Formula (2):

Autoaggregation (%) = (1 − A_t_/A_0_) × 100 (2)

where A_t_ is the absorbance at time t, and A_0_ is the absorbance at time 0.

The degree of coaggregation was determined in the same way as for assessment of autoaggregation. In this case, equal volumes of the working bacterial cell suspension and the suspension of selected test microorganisms (*Escherichia coli*, *Listeria monocytogenes*, *Staphylococcus aureus*, and *Salmonella* Typhimurium) were mixed. The absorbance at 620 nm was measured in the carefully removed upper layer of the prepared resting suspensions at intervals of 1, 2, 3, 4, and 24 h.

### 2.5. Functional Properties of A. unedo Extract Combined with Lpb. plantarum DB2

#### 2.5.1. Preparation of *A. unedo* Extract

High-power ultrasound (HPU) technology, employing a UP400St, 400 W, DN22, 24 Hz ultrasonicator (Hielscher Ultrasonics GmbH, Teltow, Germany), was used to prepare the aqueous *A. unedo* extract. The production of a high-quality extract, or an extract with a high concentration of BACs, was crucial; thus, studies were conducted beforehand to determine the optimal extraction parameters. Based on the results obtained, an optimization was performed, and the following extraction parameters were chosen: amplitude, 50%; pulse, 100%; and extraction time, 5 min; extraction temperature, 80 °C [26]. The extraction procedure was performed using the pulp of *A. unedo* (10 g ± 0.0001), to which 60 mL of distilled water was added. After extraction, the extract was filtered through Whatman filter paper No. 40 (Whatman International Ltd., Kent, UK) into a volumetric flask, adding the extraction solvent to reach a volume of 100 mL. 

#### 2.5.2. Fermentation of *A. unedo* Extract

Prior to the inoculation, *A. unedo* water extract was sterilized by filtration through 0.22 µm syringe filters. After overnight growth in MRS broth at 37 °C, the bacterial cells of the *Lpb. plantarum* DB2 culture were separated from the medium by centrifugation at 6440× *g* for 10 min. The cells were then washed with sterile deionized water and centrifuged again under the same conditions. The obtained bacterial cells were resuspended in 4 mL *A. unedo* extract with a concentration of 10^5^ CFU mL^−1^.

In sterile test tubes, 1 mL of the resulting bacterial cell suspension was mixed with 9 mL of *A. unedo* extract, fetal bovine serum as a growth propagator, and different concentrations of glucose. The test tubes were then incubated for 24 h at 37 °C under aerobic conditions. The compositions of the prepared samples are presented in Table 1.

#### 2.5.3. Antimicrobial Activity of *Lpb. plantarum* DB2 Ferment of *A. unedo* Extract

The antimicrobial activity of the fermented samples was determined as described in Section 2.4.2. Briefly, after fermentation of the A. unedo extract with the DB2 strain under different conditions (E + DB2; E + S (1%) + DB2; E + S (5%) + DB2), the samples were separated from the cells by centrifugation, followed by filter sterilization of the supernatant. Unfermented extract was added as an additional sample to the experiment, and its inhibitory activity on pathogen growth was measured.

#### 2.5.4. Freeze-Drying and Microencapsulation of Fermented and Non-Fermented *A. unedo* Extract

The fermented *A. unedo* extract was produced according to the procedure described in the previous section. After fermentation at 37 °C for 24 h, the supernatant was separated from the biomass by centrifugation at 6440× *g* for 15 min and used for the subsequent procedure.

Alginate (2%) was dissolved in 18 mL of the previously obtained supernatant of the fermented strawberry tree extract, under stirring on a magnetic stirrer. Using a medical syringe, the solution obtained was gradually added to 50 mL of a 2.5% solution of CaCl_2_ under stirring with a magnetic stirrer. After 30 min, the microcapsules formed were separated from the CaCl_2_ solution and air dried. The same procedure was repeated with an equal volume of unfermented *A. unedo* extract.

The microencapsulated non-fermented and fermented aqueous extracts of the *A. unedo* samples, each weighing 0.5 g were obtained, frozen at −80 °C, and then freeze-dried in a Christ Alpha 1-2 LD plus freeze dryer (Martin Christ Gefriertrocknungsanlagen GmbH, Osterode, Germany) for 24 h.

#### 2.5.5. DPPH Radical Scavenging of *A. unedo* Extract, before and after Microencapsulation and Freeze-Drying 

The determination of total antioxidant activity is the ability to remove free 2,2-diphenyl-1-picrylhydrazyl (DPPH) radicals, and this was determined according to the adapted method of Kostelac et al. [27]. To 5 mL of a 0.07 mM ethanol solution of DPPH, 100 µL of *A. unedo* extract or 100 µL of a fermented *A. unedo* extract was added, and the samples were homogenized. After the samples were incubated in the dark for 30 min, the absorbance was measured at 517 nm using a Helios β UV–Vis (Unicam, Cambridge, UK) spectrophotometer. The control samples contained 5 mL of an ethanol solution of DPPH and 100 µL of sterile water.

A total of 0.5 g microcapsules, containing fermented and unfermented *A. unedo* extract, were weighed, and then 4 mL of 0.07 mM ethanol solution of DPPH was added to each. The samples were briefly homogenized and incubated in the dark for 30 min. The absorbance was then measured at 517 nm. The procedure was repeated with the freeze-dried capsules. The control samples contained DPPH ethanol solution and sterile deionized water.

The percentage of DPPH free radical removal was calculated according to the following Formula (3):

Radical Removal (%) = [1 − (A − A_bl_)/A_c_] × 100 (3)

where A is the absorbance of the sample, A_bl_ is the absorbance of the blank, and A_c_ is the absorbance of the control sample.

### 2.6. Statistical Analysis

The results obtained in the study were prepared and organized using Microsoft Office Excel 2013. Statistical data analysis was performed using STATISTICA v.7.1. for Windows 10 (Stat-Soft, Tulsa, OK, USA). The ability to form biofilms was determined in four parallel experiments; antimicrobial activity and antioxidant activity after microencapsulation and freeze-drying were determined in triplicate; survivability under simulated stomach and intestinal conditions was determined in triplicate; determination of bacterial growth in water extract, antibiotic resistance, hemolytic activities, autoaggregation, coaggregation, and antioxidant activities of non-fermented and fermented extracts were performed in quadruplicate. All values are expressed as a mean ± SD. Statistical differences were determined using a t-test, with statistical significance set at *p* < 0.05.

## 3. Results and Discussion

### 3.1. Isolation and Identification of Bacterial Strains

After primary inoculation on MRS agar, the growth of diverse colonies of microorganisms was observed. Among the multitude of colonies, two milk-white colonies of round shape were observed and set apart for further isolation procedures, as their morphology corresponded to that of lactic acid bacteria.

Following the obtaining of pure cultures of these isolates, additional morphological examination was conducted, in which microscopy techniques revealed rod-shaped bacterial cells, Gram-positive after Gram staining, confirming the purity of the culture.

The two mentioned isolates, after multiplication, were labeled DB1 and DB2, and these were stored at −20 and −80 °C. 

Through the described procedures, two isolates of lactic acid bacteria, DB1 and DB2, were successfully isolated from fresh strawberry fruits and stored in the Collection of Microorganisms of the Laboratory for General Microbiology and Food Microbiology at the Faculty of Food Technology and Biotechnology, University of Zagreb, Croatia.

Using the API 50 CHL test, which is utilized for the identification of bacteria belonging to the genus *Lactobacillus*, fermentation profiles of bacterial isolates were obtained. Both isolates were identified, with a high percentage of overlap (>99%), as *Lactiplantibacillus plantarum*.

The phylogenetic analysis, based on the sequence of 16S rRNA, categorized isolated strains within the *L. plantarum* group. However, since multiple species within this group share identical 16S rRNA sequences, further investigation was conducted by analyzing the less evolutionarily conserved locus, recA. The sequence of this locus in both isolates perfectly matched that of *L. plantarum* subsp. *plantarum* (1291/1291 matches, 100% identity), distinguishing them from related species. Consequently, the sequence analysis confirmed that DB1 and DB2 are indeed *L. plantarum* subsp. *plantarum* strains.

### 3.2. Probiotic Characterization of Bacterial Isolates

#### 3.2.1. Survival of Probiotic Candidates in Simulated Gastrointestinal Fluids

**Figure 1:**
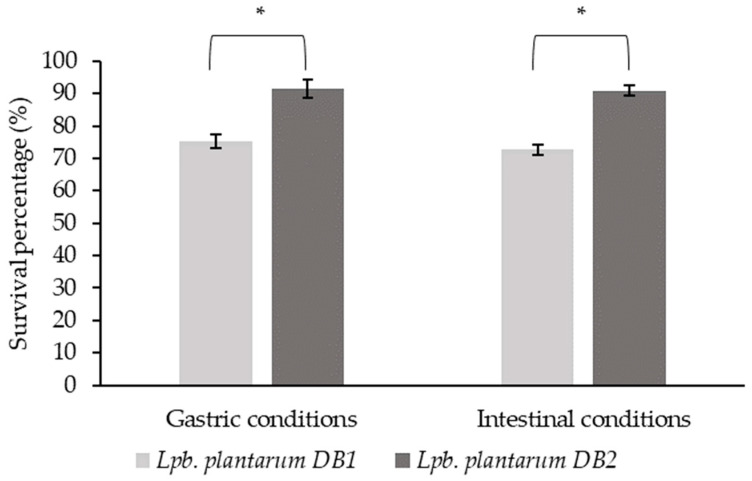
The survival of *Lpb. plantarum* DB1 and *Lpb. plantarum* DB2 under simulated gastric and intestinal conditions, expressed as a percent of survival ± SD. * Statistically significant difference at *p* ≤ 0.05.

A crucial prerequisite for the expression of the functional effects of probiotics is their ability to survive under specific conditions in different parts of the gastrointestinal tract in order to reach the target site in the host organism. Therefore, the ability of isolates DB1 and DB2 to survive under simulated conditions of the stomach and intestine was investigated in this study. The results are shown in Figure 1.

According to the results obtained, the strains showed a moderate to high survival rate under simulated stomach and intestinal conditions, with a survival rate over 70% for the DB1 strain in both cases and over 90% for the DB2 strain in both cases (Figure 1). These results are consistent with the results of other studies, such as the one conducted by Nath et al. [28], in which the survival rate of the *L. plantarum* strain tested was over 90%.

The viability of probiotics in the gastrointestinal tract and their delivery to the target site in the body is the essential prerequisite for exerting a probiotic effect. Based on this fact, *L. plantarum* DB2, which displayed a greater than 90% survival rate, was selected for future experiments.

#### 3.2.2. Antimicrobial Activity of *Lpb. plantarum* DB2 against Pathogenic Bacteria

**Figure 2:**
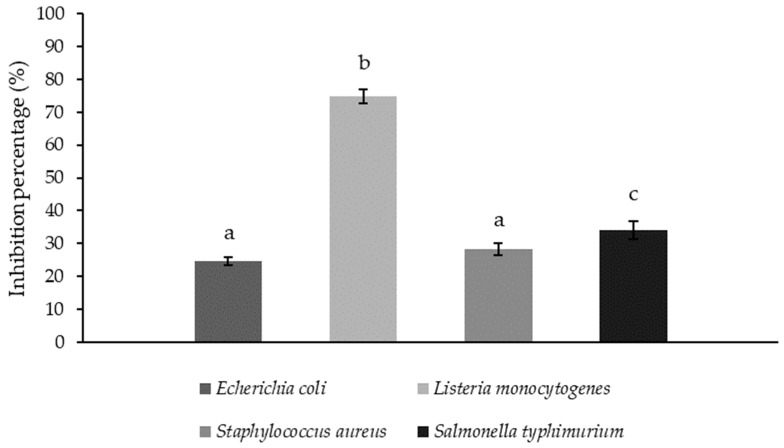
Antimicrobial activity of *Lpb. plantarum* DB2 supernatant against common pathogenic bacteria presented as an inhibition percentage of the control pathogen growth ± SD. ^a,b,c^—different letters designate a statistically significant difference at *p* ≤ 0.05.

Since the inhibitory impact of potential probiotics against pathogenic microorganisms is a fundamental prerequisite for the development of positive effects, the antimicrobial activity of strain DB2 against common pathogenic microorganisms was investigated in this study. The growth of pathogens in the presence of the supernatant of the tested strain cultured under different conditions was monitored, and the antimicrobial activity was determined using the turbidimetric method. The results are shown in Figure 2.

The bacterial strain *Lpb. plantarum* DB2 exhibited varying inhibition effects against pathogenic microorganisms under all tested growth conditions. 

The highest inhibitory activity for the DB2 supernatant, with a pH of 3.88 ± 0.04, was determined against *L. monocytogenes*, which was about 75% that of the control. 

One of the fundamental functional properties of probiotics is their antimicrobial activity against pathogenic microorganisms, which manifests itself through various mechanisms, such as the secretion of bacteriocins and other compounds with antimicrobial activity, or through food competition, ultimately aiming to protect the organism from pathogens [29]. Studies by Jeong et al. [30] have demonstrated the antimicrobial activity of various *Lpb. plantarum* strains, with inhibition thresholds ranging from 15% to 23% against pathogens such as *E. coli*, *L. monocytogenes*, and *S. aureus*. The probiotic properties depend solely on the strain and can vary considerably between bacteria of the same species [31]. Therefore, differences in the inhibitory effects of lactic acid bacteria of the same genus and species are not unexpected, as shown in the study by Hu et al. [32]. Furthermore, lowering the pH through lactic acid production is one of the main inhibitory mechanisms of lactic acid bacteria and could be the key inhibitor in this study. Further research should focus on investigating the possibility of other antimicrobial effectors produced by the DB2 strain. 

#### 3.2.3. Antibiotic Resistance Assay

Antibiotic resistance poses a health problem for the host, as it can be transferred to pathogenic bacteria. Therefore, evaluating the antibiotic resistance of potential probiotic bacteria is an important step to ensure their safety. In this study, the susceptibility of the DB2 strain to selected antibiotics was investigated using the disk diffusion method. The strain was categorized as sensitive, moderately sensitive, or resistant, based on the diameter of the inhibition zones.

According to the results obtained, the tested isolate showed resistance (no visible inhibition zones after incubation) to the same antibiotics, i.e., kanamycin, gentamicin, and streptomycin, which belong to the group of aminoglycoside antibiotics, as well as vancomycin. The DB2 isolate showed clear inhibition zones (sensitivity) for all other tested antibiotics. Resistance to vancomycin is widespread in certain bacterial strains of the genus *Lactobacillus,* as demonstrated by Zhou et al. [33] in their research. They found that vancomycin resistance is an intrinsic, chromosomally encoded trait that is not transferable to other bacterial strains and therefore, does not pose a potential risk. The authors of the same study also observed resistance to aminoglycoside antibiotics in *Lpb. plantarum* strains, which is also considered an intrinsic bacterial trait and does not pose a risk for transmission of resistance to other bacteria [34]. The transfer of resistance is mainly mediated by plasmids carrying antibiotic resistance genes, whereas intrinsic resistance does not pose a threat, but can be beneficial for the host [33]. Further studies are required to confirm that the DB2 strain is safe for food applications (whole genome sequencing and the study of the genes involved in the resistance to antibiotics).

#### 3.2.4. Hemolytic Activity

One of the basic safety criteria for the selection of probiotic bacteria is that they do not exhibit hemolytic activity, i.e., the ability to lyse red blood cells, which leads to the release of their contents into the bloodstream, which can have harmful consequences for human health [35]. For this reason, the hemolytic activity of the tested DB2 strain was investigated. The results showed that there were no visible zones noted in the blood agar, a condition which is classified as γ-hemolysis. This means that the strain tested has no hemolytic activity and is considered safe for human use, according to this criterion. The absence of hemolytic activity is consistent with the results from other literature sources indicating that *Lpb. plantarum* strains do not exhibit hemolytic activity [36].

#### 3.2.5. Biofilm Formation Ability

A comprehensive probiotic characterization also includes the investigation of the ability of probiotic candidates to form biofilms. In this study, the ability of DB2 to form biofilms was investigated and categorized as weak, moderate, or strong. The results of biofilm formation after 24 h and 48 h for the tested isolate are shown in Table 2.

According to the results presented in Table 2, the DB2 strain is classified as a strong biofilm producer in both cases (24 and 48 h). Based on the change in optical density between 24 and 48 h, we can conclude that incubation time significantly affects biofilm formation, and a higher value was obtained after 48 h for the strain tested.

The ability to form biofilms, complex bacterial communities linked by the extracellular matrix they produce, can significantly influence the functionality of probiotic bacteria. It improves their survival under gastrointestinal conditions, the colonization of the gastrointestinal tract, and their competitiveness against pathogenic species. It can also stimulate the production and release of various active molecules important for host health, when compared to the abilities of individual (planktonic) cells [37]. Biofilm formation is directly related to adhesion capability, which is crucial for the competitive exclusion of pathogenic microorganisms [38]. The results of this study are in agreement with those of Iorizzo et al. [39], whose research showed that all five tested strains of *Lactiplantibacillus plantarum* have the ability to form biofilms, although this ability is highly strain-dependent. Rezaei et al. [40] also showed the impact of incubation time on the strength of biofilm formation.

Based on the results of this study and the aforementioned literature results, it can be concluded that the strain *Lpb. plantarum* DB2 has a strong ability to form biofilms, indicating its ability to form biofilms in the host gastrointestinal system and its potential as a probiotic.

#### 3.2.6. Autoaggregation and Coaggregation of *Lpb. plantarum* DB2

In addition to the ability to form biofilms, autoaggregation and coaggregation are other probiotic criteria related to the colonization of the gastrointestinal tract. Since the strain *Lpb. plantarum* DB2 demonstrated a high level of survival and a strong ability to form biofilms under simulated gastrointestinal tract conditions, its ability to autoaggregate and coaggregate with common pathogenic microorganisms was determined (Table 3).

The values of coaggregation of the strain *Lpb. plantarum* DB2 with the tested pathogens, expressed as the percentage of coaggregated cells relative to the initial value, increased with time and reached the highest values after 24 h of incubation, ranging from 24.76% to 63.01%. The lowest coaggregation was seen in *S.* Typhimurium, while the highest was seen in *S. aureus*.

Autoaggregation (the ability of bacterial cells to aggregate with each other) facilitates better adhesion in the gastrointestinal tract and promotes colonization [41,42,43]. The coaggregation of probiotics with pathogenic cells contributes to the antimicrobial effect of probiotics against pathogens and has a positive effect on the reduction of gastrointestinal colonization by pathogenic microorganisms [44]. The autoaggregation values obtained in this study are significantly higher than those reported by Guan et al. [45] for the tested *Lpb. plantarum* BXM2 isolate (93.99% compared to 55.41%). The coaggregation results align with those of other studies, such as Guan et al. [45], in which the coaggregation values of the tested *Lpb. plantarum* BXM2 isolate with the pathogens *E. coli*, *S. aureus*, and *S.* Typhimurium ranged from 34.05% to 58.34%.

### 3.3. Functional Properties of A. unedo Extract Combined with Lpb. plantarum DB2

#### 3.3.1. Fermentation of *A. unedo* Extract

Fermentation of various foods can yield a wide range of functional products that offer significant health benefits, such as reducing the risk of cardiovascular disease [46] and certain types of cancer [47], protection against infections, and immunomodulatory effects [48]. Against this background, this study explored the potential of aqueous *A. unedo* extract fermented with *Lpb. plantarum* DB2 for the development of new functional probiotic products. 

To extend the shelf life and preserve the probiotic properties of the resulting product, both non-fermented and fermented strawberry tree aqueous fruit extracts were microencapsulated and freeze-dried. The antioxidant activity of the obtained samples was determined as a measure of the success of the processes and the preservation of the probiotic properties.

**Figure 3:**
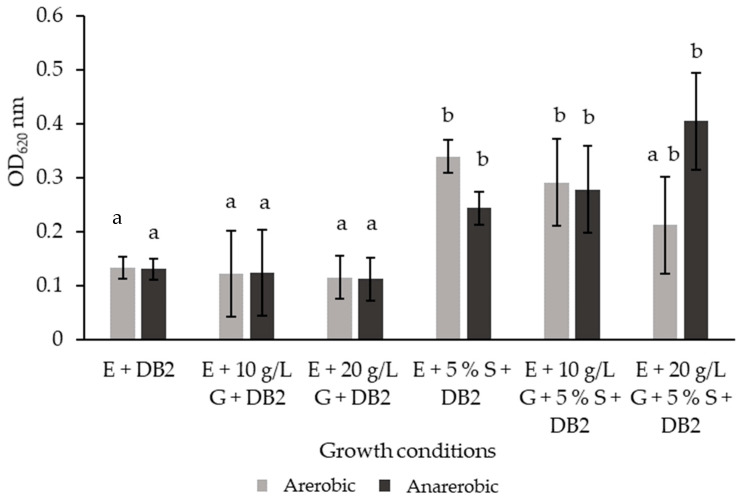
The growth ability of the *Lpb. plantarum* DB2 strain in aqueous *A. unedo* extract, with added serum and varying concentrations of glucose, under aerobic and anaerobic conditions, presented as optical density at 620 nm ± SD. E—*Arburus unedo* extract; G—glucose; DB2—LAB strain; S—fetal bovine serum. ^a,b^—different letters designate a statistically significant difference at *p* ≤ 0.05.

For a fermentation process to be successful and result in a product with the desired technological and functional properties, it is important that the selected probiotic strain has the ability to grow and survive in the chosen fermentation medium. Therefore, in this study, the growth ability of the DB2 strain in aqueous *A. unedo* extract, with and without the addition of nutrients (glucose and fetal bovine serum), was investigated under aerobic and anaerobic conditions during a 24 h incubation. The results are presented in Figure 3.

The results obtained show that the growth of the tested isolate in pure strawberry tree fruit extract was inhibited (Figure 3). The results also show that the addition of glucose had no effect on bacterial growth. Only after the addition of serum was growth significantly increased. Based on the results obtained, we hypothesize that the inhibited growth in the extracts is due to the lack of essential growth promoters, which are abundant in fetal bovine serum. It is also important to mention that the extracts of *A. unedo* exhibit high antioxidant activity, which is mainly due to their high composition of polyphenols and vitamins [4]. Such extracts have shown high antimicrobial activity in the past [6,49], but in this study, the addition of serum significantly increased the growth of the strain used, so we can conclude that the extract composition did not completely inhibit the growth of the probiotic strain used in this study. Furthermore, there was no statistically significant difference between aerobic and anaerobic conditions in the pure extract. The highest growth was only recorded under anaerobic conditions after the addition of 5% serum. Although fruit extracts represent a significant potential for the development of functional products, there are currently few studies in the literature on the topic of fermented *A. unedo* extracts and their functional benefits. As stated above, this could be due to the composition of the extracts, resulting in a limited growth medium for microorganisms. A similar fermentation in aqueous fruit extract was noted in the research of Mahmoudi et al. [50], which showed that the *L. plantarum* strain reached maximum growth levels after 24 h, entering a stationary phase and a death phase after 40 h of fermentation in an aqueous jujube (*Ziziphus jujube*) extract. Due to the lack of literature sources, further studies are needed to determine the optimal growth conditions for probiotics in fruit extracts, as well as the benefits of such fermentation for the biological properties of extracts, with the possibility of developing functional products with the synergistic functional aspects of probiotics and active fermented extracts. 

#### 3.3.2. Antimicrobial Activity of *Lpb. plantarum* DB2 Ferment of *A. unedo* Extract

**Figure 4:**
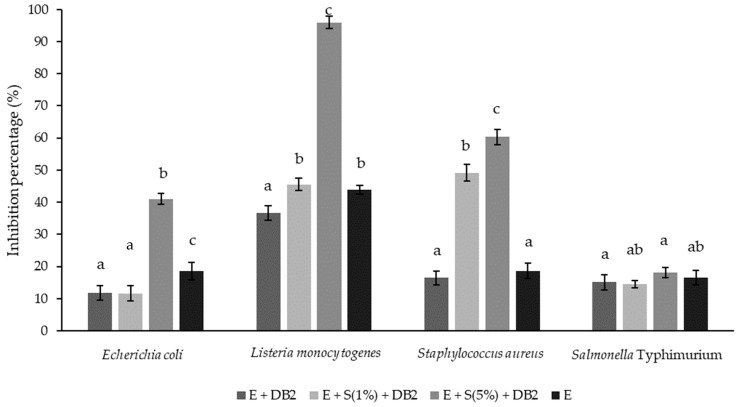
The antimicrobial activity of the of *Lpb. plantarum* DB2 ferment of *A. unedo* extract against selected pathogens expressed as a percent of growth inhibition ± SD. ^a,b,c^—different letters designate statistically significant differences between the same pathogen at *p* ≤ 0.05. E—aqueous *A. unedo* extract; S—fetal bovine serum. Samples: E + DB2 (extract fermented with DB2); E + S (1%) + DB2 (extract fermented with DB2 in the presence of serum 1% *v*/*v*); E + S (5%) + DB2 (extract fermented with DB2 in the presence of serum 5% *v*/*v*); E (unfermented extract).

In order to investigate the functional potential of fermented extracts, their antimicrobial activity against common pathogenic bacteria was determined. The inhibitory potential towards pathogens was determined in the presence of the *Lpb. plantarum* DB2 ferment of *A. unedo* extract, with the addition of serum as a growth propagator in two concentrations, compared to that noted for unfermented extract and the ferment with the unstimulated growth of DB2 (no serum). The results are presented in Figure 4.

The results indicate that the highest inhibition of *E. coli*, *L. monocytogenes*, and *S. aureus* occurred in the presence of the ferment obtained in the sample in which DB2 growth was stimulated by the addition of 5% serum. *Salmonella* was moderately inhibited, with no significant difference between the fermented and non-fermented samples. Strawberry tree extract alone showed a significantly lower inhibition of all pathogens, with the exception of *Salmonella*, compared to that of the 5% serum-induced DB2 in the same extract. Important aspects of the obtained results can be noted from the comparison of Figure 2 and Figure 4. Comparing this data indicates that the fermentation with DB2 (stimulated with 5% serum) significantly increased the inhibition of *E. coli*, *L. monocytogenes*, and *S. aureus* compared to that of the DB2 and the extract individually, thus highlighting the possibility of synergistic functional effects. *A. unedo* root water extracts have previously demonstrated antimicrobial activity against *E. coli* and *S. aureus*, with this activity attributed to a high polyphenol concentration [51]. Varying antimicrobial activities were documented by leaf water extracts [52]. To our knowledge, there are still no published studies that investigate the antimicrobial activity of *A.unedo* fruit water extract fermented with lactic acid bacteria. 

#### 3.3.3. DPPH Radical Scavenging of *A. unedo* Extract before and after Microencapsulation and Freeze-Drying 

**Figure 5:**
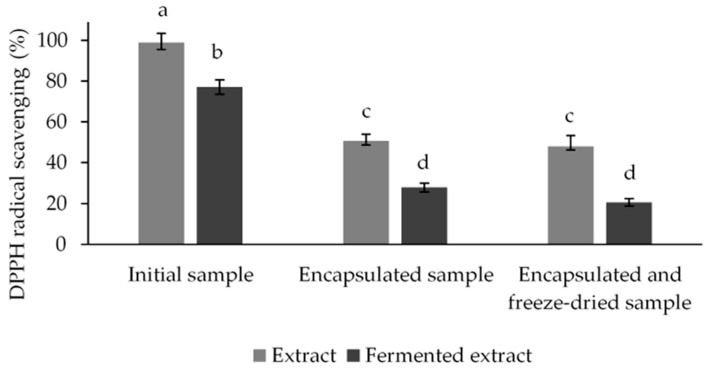
DPPH scavenging ability of fermented and non-fermented aqueous *A. unedo* extracts and their microencapsulates before and after freeze-drying, presented as a percent ± SD, with a statistically significant difference at *p* ≤ 0.05. ^a,b,c,d^—different letters designate statistically significant difference at *p* ≤ 0.05.

Antioxidant activity is one of the most important and most researched functional properties of functional foods. Therefore, in this study, the antioxidant activity of unfermented aqueous *A. unedo* extract fermented with the *Lactiplantibacillus plantarum* DB2 strain and their microencapsulates, before and after freeze-drying, was investigated. The total antioxidant activity was determined as the ability to eliminate DPPH free radicals and was expressed as a percentage of DPPH radicals eliminated (Figure 5).

The results shown in Figure 5 indicate that the aqueous *A. unedo* extract has a strong antioxidant capacity, which decreased after fermentation with the bacterial culture. The ability to remove DPPH free radicals decreased from the initial 98.98% to 77.15% after fermentation. Freeze drying preserved the antioxidant activity of the microencapsulated samples, with a slight decrease from the initial 50.72% to 48.05% for the non-fermented aqueous *A. unedo* extract and from 27.81% to 20.54% for the fermented aqueous *A. unedo* extract. In all three cases, the non-fermented aqueous *A. unedo* extract exhibited better antioxidant activity than did the fermented extract. The freeze-drying process can significantly contribute to extending the shelf life of probiotic products and the survival of probiotic cultures, while preserving their functional properties [53]. In this study, after microencapsulation and freeze-drying processes, significant antioxidant activity was still observed in the tested samples, at about 50% for the non-fermented aqueous *A. unedo* extract and over 20% for the fermented aqueous *A. unedo* extract. We hypothesize that the LAB fermenting activity reduced radical scavenging potential due to the metabolism of polyphenolic compounds. The obtained results are limited by the use of only one method to measure antioxidant potential, so a more detailed investigation of this activity should be conducted in future studies. Additionally, microencapsulation in alginate beads could reduce the availability of radical scavenging components in the employed assay, so future studies will be directed to elucidate the retained functional activity (antimicrobial and antioxidant) after the natural degradation of alginate beads under intestinal conditions and optimize and reduce the loss of antioxidant activity during these processes.

## 4. Conclusions

In this study, the *Lactiplantibacillus plantarum* DB2 strain isolated from the strawberry was successfully probiotically characterized, meeting the safety criteria for use in the production of functional foods. The antimicrobial activity varied with the cultivation conditions and showed a higher inhibition in fermented strawberry tree extract with serum added as growth propagator. DB2 exhibited a robust survival rate (>90%) under simulated gastrointestinal conditions, high biofilm formation, autoaggregation (>90% after 24 h), and coaggregation with pathogens. Fermentation of the aqueous *A. unedo* extract with DB2 retained antioxidant activity at level of >70%, along with increased antimicrobial potential. Microencapsulation resulted in a significant reduction of radical scavenging potential for both the fermented and non-fermented extracts, and freeze-drying had no effect on antioxidant activity. This underlines the potential for the development of new functional products by combining fruit substrates with isolated probiotics. As this work shows, there is a potential application for fruit-derived probiotics in the fermentation of fruit extracts, leading to combined probiotic–extract products with a wider range of biological activities, but future studies are required to better identify the fermentation dynamics and mechanisms of action in such complex systems.

## Figures and Tables

**Table 1:** *A. unedo* water extract fermentation with *Lpb. plantarum* DB2 sample composition.

Sample	Composition
E + DB2	Extract inoculated with DB2 strain
E + G (1%) + DB2	Extract with 1% *w*/*v* glucose inoculated with DB2 strain
E + G (2%) + DB2	Extract with 2% *w*/*v* glucose inoculated with DB2 strain
E + S (5%) + DB2	Extract with 5% *v*/*v* serum inoculated with DB2 strain
E + G (1%) + S (5%)+ DB2	Extract with 1% *w*/*v* glucose and 5% *v*/*v* serum inoculated with DB2 strain
E + G (2%) + S (5%)+ DB2	Extract with 2% *w*/*v* glucose and 5% *v*/*v* serum inoculated with DB2 strain

**Table 2:** The ability of bacterial strain *Lpb. plantarum* DB2 to form biofilms is expressed as the mean optical density (OD) ± standard deviation (SD), expressed in percent ± SD, with a statistically significant difference of *p* ≤ 0.05.

		24 h		48 h	
		OD_595_ nm	Classification	OD_595_ nm	Classification
Reference Values ^#^	ODC	0.127 ± 0.01	/	0.073 ± 0.01	/
	2 × ODC	0.254 ± 0.01		0.147 ± 0.01	
	4 × ODC	0.508 ± 0.01		0.293 ± 0.01	
Tested strain	*Lpb. plantarum* DB2	0.522 ± 0.17	Strong formation	0.715 ± 0.11 *	Strong formation

OD—optical density at 595nm; ODC–optical density of the control. * Statistically different from 24 h sample. ^#^ Reference values were set according to those of Borges et al. [23].

**Table 3:** The ability of bacterial strain *Lpb. plantarum* DB2 to autoaggregate and coaggregate with common pathogens at time intervals greater than 24 h of incubation is presented in percent ± SD, with a statistically significant difference at *p* ≤ 0.05.

**Autoaggregation (%)**				
Time (h)	*Lactiplantibacillus plantarum DB2*			
1	1.34 ± 0.21			
2	4.71 ± 0.35			
3	5.47 ± 0.81			
4	5.52 ± 0.98			
24	93.87 ± 1.23			
**Coaggregation (%)**				
Time (h)	*S.* Typhimurium + DB2	*E.coli* + DB2	*S. aureus* + DB2	*L. monocytogenes* + DB2
1	4.26 ± 0.45	1.82 ± 0.21	2.03 ± 0.45	3.18 ± 0.21
2	4.23 ± 0.32	1.45 ± 0.32	4.07 ± 0.32	6.37 ± 0.32
3	6.51 ± 0.67	2.55 ± 0,91	6.10 ± 0.71	3.82 ± 0.98
4	6.84 ± 1.31	4.73 ± 1.51	6.50 ± 2.31	5.73 ± 1.78
24	24.76 ± 1.42 ^a^	27.27 ± 2.62 ^a^	63.01 ± 2.01 ^b^	35.67 ± 1.23 ^c^

^a,b,c^—different letters designate statistically significant difference between groups after 24 h at *p* ≤ 0.05.

## Data Availability

The raw data supporting the conclusions of this article will be made available by the authors on request.
